# Ceramic-Reinforced γ-TiAl-Based Composites: Synthesis, Structure, and Properties

**DOI:** 10.3390/ma12040629

**Published:** 2019-02-20

**Authors:** Daria V. Lazurenko, Andreas Stark, Maksim A. Esikov, Jonathan Paul, Ivan A. Bataev, Adelya A. Kashimbetova, Vyacheslav I. Mali, Uwe Lorenz, Florian Pyczak

**Affiliations:** 1 Novosibirsk State Technical University, Karl Marks str. 20, 630073 Novosibirsk, Russia; esmax@ya.ru (M.A.E.); ivanbataev@ngs.ru (I.A.B.); k.yaleda@mail.ru (A.A.K.); 2 Helmholtz Zentrum Geesthacht, Max-Planck-Straße 1, 21502 Geesthacht, Germany; andreas.stark@hzg.de (A.S.); jonathan.paul@hzg.de (J.P.); uwe.lorenz@hzg.de (U.L.); florian.pyczak@hzg.de (F.P.); 3 Lavrentiev Institute of Hydrodinamics, SB RAS, Lavrentiev av. 15, 630090 Novosibirsk, Russia; vmali@mail.ru

**Keywords:** composite materials, intermetallics, sintering, microstructure, phase transitions, synchrotron radiation

## Abstract

In this study, new multilayer TiAl-based composites were developed and characterized. The materials were produced by spark plasma sintering (SPS) of elemental Ti and Al foils and ceramic particles (TiB_2_ and TiC) at 1250 °C. The matrix of the composites consisted of α_2_-TiAl and γ-TiAl lamellas and reinforcing ceramic layers. Formation of the α_2_ + γ structure, which occurred via a number of solid–liquid and solid–solid reactions and intermediate phases, was characterized by in situ synchrotron X-ray diffraction analysis. The combination of X-ray diffraction (XRD), transmission electron microscopy (TEM), scanning electron microscopy (SEM), and energy dispersive X-ray (EDX) analysis revealed that an interaction of TiC with Ti and Al led to the formation of a Ti_2_AlC M_n+1_AX_n_ (MAX) phase. No chemical reactions between TiB_2_ and the matrix elements were observed. The microhardness, compressive strength, and creep behavior of the composites were measured to estimate their mechanical properties. The orientation of the layers with respect to the direction of the load affected the compressive strength and creep behavior of TiC-reinforced composites. The compressive strength of samples loaded in the perpendicular direction to layers was higher; however, the creep resistance was better for composites loaded in the longitudinal direction. The microhardness of the composites correlated with the microhardness of reinforcing components.

## 1. Introduction

The development of new materials for gas turbine engines is currently one of the key areas in the field of power and airspace engineering. The traditional materials for application in this field are iron–nickel and cobalt-based alloys, which are famous due to their high oxidation resistance, high-temperature strength, and good creep characteristics [1]. However, the main disadvantage of these materials is their high density. An efficient way to decrease the weight of structural elements for aircraft applications consists in the replacement of heavy alloys by intermetallics that contain aluminum. Nickel aluminide (Ni_3_Al) is a good example of such a compound that could be used for high-temperature applications [2]. Ni_3_Al-based superalloys are recommended for the fabrication of gas turbine engines running in the temperature range between 800 and 1000 °C. However, the application of these materials does not result in significant weight reductions [3]. For this reason, titanium aluminides, having a much lower density, are currently considered to be better candidates than heavier Ni_3_Al-based alloys [4]. The α_2_-Ti_3_Al and γ-TiAl alloys are characterized by such properties as high specific strength, specific stiffness, and oxidation resistance at elevated temperatures. They can be used in applications up to 700 °C and 900 °C, respectively [5]. Thus, the investigation and application of γ-TiAl is currently an active research area. At present, γ-TiAl-based alloys are used for the fabrication of turbine blades, automobile valves, and turbocharger wheels [6,7,8,9,10].

The main disadvantage of intermetallic alloys based on titanium aluminides is their low temperature brittleness (the room temperature ductility does not exceed 2%), which makes the workability of these alloys very difficult.

However, the microstructure and properties of the alloys based on the mixture of α_2_ and γ phases can be controlled by varying the Al/Ti ratio and selection of heat treatment regimes [7,11,12,13]. Such alloys normally contain between 40 and 48 atomic% Al. Lower Al-containing binary alloys have increased strength, but at the same time reduced room temperature ductility and oxidation resistance compared to the alloys with the highest possible Al content. Alloying additions of up to 2 at.% of Cr, Mn, or V improves the room temperature ductility; the oxidation resistance as well as high-temperature strength of (α_2_ + γ) alloys can be improved by alloying with 1–3 at.% of Nb, Ta, Mn, Zr, Hf, and W; titanium diboride refines grains and can increase ductility and also high-temperature strength due to precipitation hardening [13].

An alternative way to potentially improve the strength of γ-TiAl materials (not related to alloying and heat treatment) is fabrication of intermetallic-based composites. Combination of dissimilar materials could be a reasonable approach to obtain an outstanding combination of mechanical properties. In a number of studies [14,15,16,17,18], the possibility of improving physical, chemical, and mechanical properties of titanium aluminides by reinforcement with hard particles has been considered. In most cases, a reinforcing phase was homogeneously distributed in the intermetallic matrix. TiC [18,19,20], Ti_2_AlC [20,21,22], TiB_2_ [23], and SiC [24,25] as well as different oxides [18] and nitrides [26,27] have been used as a reinforcement phase. For instance, Yue et al. [19] showed that the addition of 5 vol% TiC to γ-TiAl alloy increased its crack grow resistance by almost 45%. The maximum strength was reached with 10 vol% of reinforcing particles. Increasing the volume fraction of hard particles in the structure of the material (up to 20–40%) positively influenced the wear resistance of the intermetallic material. A γ-TiAl—TiB_2_ composite has been reported to have high strength characteristics both at the room and elevated temperatures, with the maximum strength being observed for 10 vol% of the reinforcement phase [23].

Properly designed and utilized laminated composites can greatly improve the mechanical performance of structural parts. Over the last few decades, a number of studies were devoted to the fabrication and characterization of metallic- and intermetallic-based multilayer composites. These materials typically consist of various similar and dissimilar metals and intermetallic compounds [28,29,30]. Complex composite structures containing metallic and intermetallic layers with ceramic particles and fibers have also been recently developed [31,32,33,34,35]. Multilayered materials possess a unique combination of mechanical properties. Among them, the fracture toughness as well as the impact and ballistic properties are particularly impressive [36]. The resistance to ballistic impact can be explained by local delamination, which reduces the stiffness of individual layers. This allows them to bend and increases the volume of material that absorbs the energy released during impact. The fracture and impact toughness increase due to the action of such mechanisms as crack deflection, crack blunting and bridging, and stress redistribution during delamination. The other important feature of multilayer composites is the significant anisotropy of properties. Li et al. [37] have shown that the compressive strength of metallic–intermetallic laminated composites can differ significantly in different loading directions (i.e., normal or parallel to the layers). Thus, for specific loading conditions, the optimal orientation of the multilayer within the material should be known to achieve the best performance.

In this study, the two aforementioned approaches have been combined to fabricate a novel intermetallic-based material. Intermetallic matrix laminated composites with layers of either TiC or TiB_2_ ceramic particles, as the reinforcing phases, have been fabricated. The composite was produced using a reactive foil sintering technique via a series of complex solid–liquid and solid–solid reactions. The aim of this study is to investigate the phase transformations that occur during synthesis of the intermetallic phases from the elemental foils and the characterization of the structure and properties of the produced ceramic-particle-reinforced γ-TiAl-based composites.

## 2. Materials and Methods

**Figure 1:**
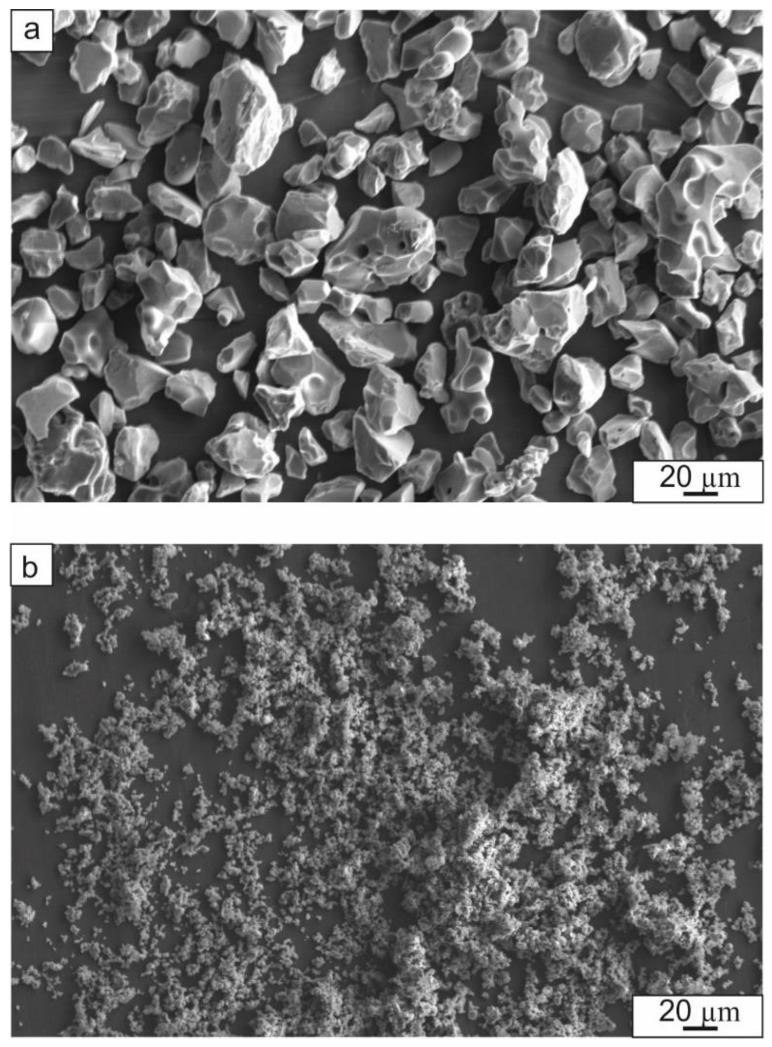
SEM images of reinforcing particles: (**a**) TiB_2_; (**b**) TiC.

**Figure 2:**
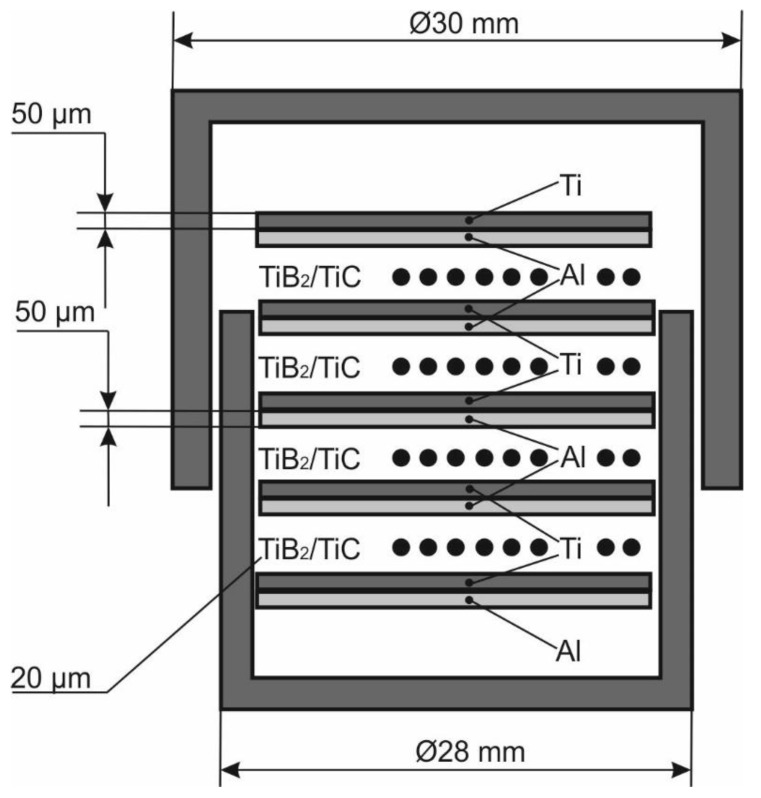
The scheme of the workpiece for sintering.

Titanium (99.2% Ti, 0.18% Fe, 0.06% C, 0.1% Si, 0.04% N, 0.12% O) and aluminum (99.5% Al, 0.26% Fe, 0.3% Si, 0.03% Ti, 0.02% Cu, 0.06% Zn) foils both with a thickness of 50 µm were used to synthesize the intermetallic TiAl. TiB_2_ or TiC powders were chosen as the reinforcement phases (Figure 1). Figure 2 shows schematically how the samples were prepared. Titanium and aluminum foils as well as the reinforcing powder were alternatively placed in a titanium container with an outer diameter of 28 mm and an inner diameter of 26 mm. The weight of TiB_2_ or TiC powders used for one layer were 0.047 g and 0.052 g, respectively. These masses of powders should provide the thickness of the ceramic layers equal to 20 µm after sintering. The titanium container was covered by a titanium cap with an outer diameter of 30 µm. The use of a titanium container prevented leakage of molten aluminum during the subsequent sintering of samples.

The titanium containers with the arrangement of foils and powder inside were further placed within a graphite die and sintered using a Labox 1575 spark plasma sintering (SPS) machine (SINTER LAND Inc., Nagaoka, Japan). The sintering procedure was carried out in two steps. During the first step, the samples were heated up to 830 °C and held at this temperature for 10 min. The pressure applied during this step was 40 MPa. As was shown in [38], this heating period was sufficient to consume the layers of aluminum and obtain a composite consisting of titanium and titanium trialuminide phases. During the second step, the temperature was increased to 1250 °C. At this temperature, the reaction between titanium and titanium trialuminide is expected to result in the formation of titanium-rich intermetallic compounds. This temperature was maintained for 5 min; with the pressure being decreased to 10 MPa. During the sintering, the temperature was measured by a thermocouple mounted in the graphite die. The heating rate was equal to 50 °C per minute, and the cooling rate was 100 °C per minute.

The microstructure of samples after the first and the second steps of sintering was investigated by scanning electron microscopy (SEM) using a LEO Gemini 1530 microscope (Carl Zeiss AG, Oberkochen, Germany) equipped with an Octane Plus energy dispersive X-ray detector (AMETEK EDAX, Mahwah, NJ, USA). The specimens for microscopy were prepared according to the following procedure. The samples were cut using a Sodick AG400L wire discharge machine (Sodik Co., Ltd., Yokohama, Japan). Then, the cross section of specimens was ground using abrasive paper (up to P2500) and polished using a VibroMet 2 vibropolishing machine (Buehler, Lake Bluff, Illinois, IL, USA). The fine structure was investigated using a Philips CM200 transmission electron microscope (TEM) (Philips-FEI, Hillsboro, OR, USA). The specimens for observation in the TEM were prepared by grinding using a Gatan Model 656 dimple grinder (GATAN, Pleasanton, CA, USA) and ion polished in a Gatan 691 precision ion polishing system (GATAN, Pleasanton, CA, USA). The average phase composition of materials was studied using synchrotron X-ray diffraction. The phases in the local areas were determined using electron diffraction in the TEM. 

Synchrotron X-ray radiation diffraction was also used to observe the sequence of reactions between pure Ti and Al leading to the formation of a γ-TiAl-based material via several solid–liquid and solid–solid reactions. For this experiment, commercially pure Ti and Al powders were used. They were mixed in equiatomic proportion and placed in a thin cylindrical Ti container, which was closed with a Ti cup welded on top of it. The sample was then placed in an induction furnace, that was mounted in a DIL805A/D dilatometer, and heated with a rate of 10 °С per minute. At 830 °С, the sample was held at temperature for 10 min, and then heating was continued up to 1250 °С where the temperature was held for 5 min. Thus, the heating temperature and the holding time were the same as during SPS. Cooling was carried out at a rate of 50 °С per minute. The temperature was measured by an S-type thermocouple welded to a container wall. The experiment was performed in a high-purity argon atmosphere at a pressure of 0.8 mbar. To minimize the influence of oxygen on the powder mixture, the chamber with the mounted powder compact sample was evacuated three times to a pressure of 2 × 10^−4^ mbar using a turbomolecular pump and flushed with argon. Diffraction patterns were obtained at the Petra III synchrotron radiation source of the German Electron Synchrotron (Deutsches Elektronen-Synchrotron—DESY, Hamburg, Germany) in the High Energy Materials Science Beamline (P07) operated by Helmholtz-Zentrum Geesthacht. The X-ray radiation had an energy of 100 keV, which corresponds to a wavelength of 0.124 Å. The spot size was 1 × 1 mm. Diffraction rings were recorded in the transmission mode using a Perkin Elmer XRD1621 2D detector (Perkin–Elmer Corp., Waltham, MA, USA) with a resolution of 2048 × 2048 pixels and a pixel size of 200 × 200 µm. The sample-to-detector distance was 1837 mm. The Debye–Scherrer diffraction rings were continuously recorded during the heating, holding, and cooling stages of the experiment with a frequency of 0.1 Hz. The total exposure time for each frame was 4 s, which was obtained from the summation of 40 frames each exposed for 0.1 s. Two-dimensional diffraction rings were azimuthally integrated and analyzed as normal intensity against 2θ powder diffraction patterns.

Mechanical properties of local areas within the multilayered materials were estimated from microindentations made using a Wolpert Group 402 MVD microhardness tester (Wilson Wolpert Instruments, Aachen, Germany). The load on the diamond pyramid tip was 0.2 kg. Five measurements of each layer were done for statistical purposes. To investigate the influence of the reinforcement phase on high-temperature properties, creep tests were performed using SATEC mentor M3 testing equipment (Instron Industrial Products Group, Grove City, PA, USA). Cylindrical specimens with a diameter of 2 mm and a length of 3 mm were used for testing. A compressive load was applied to the samples in a direction that was either parallel or perpendicular to the foils. The creep samples were loaded to 250 MPa stress at 750 °С in the air atmosphere. The room temperature strength of the materials was determined by compression testing of five specimens per point using an Instron 3369 testing frame. The cubic specimens with dimensions of 5 × 5 × 5 mm^3^ were used for testing. The loading rate was 10 mm per minute.

## 3. Results and Discussion

### 3.1. Observation of the Structural Transformations in the Ti–Al System Using in Situ Synchrotron Diffraction Analysis

Fabrication of Ti–Al-based intermetallic laminated composites can be carried out via solid–solid [39] or solid–liquid processes [38]. In the latter case, the process can be accomplished much faster [38]. However, the main problem of the solid–liquid processes is the possible leakage of molten aluminium from the reaction zone even at low pressures or when no pressure is applied. This problem was addressed by Lazurenko et al. [38]. The authors demonstrated that the application of Ti containers for sintering prevented squeezing of the liquid phase and that the Al was completely consumed in the formation of intermetallic compounds. Thus, based on this previous experience, it was decided to carry out pre-sintering in titanium containers at 830 °С (for 10 min) in order to transform the Al to an Al_3_Ti phase via a solid–liquid reaction. The formation of an α_2_+γ structure was expected to take place during the second stage (1250 °С, 5 min) via the solid-state reaction of the Al-rich intermetallic phases with pure Ti.

To obtain a better understanding of the foil sintering process, in situ synchrotron diffraction analysis during the heating of elemental Ti and Al powders was carried out. The aim of this experiment was to investigate the processes occurring during the sintering of Ti and Al in order to optimize the final phase composition.

**Figure 3:**
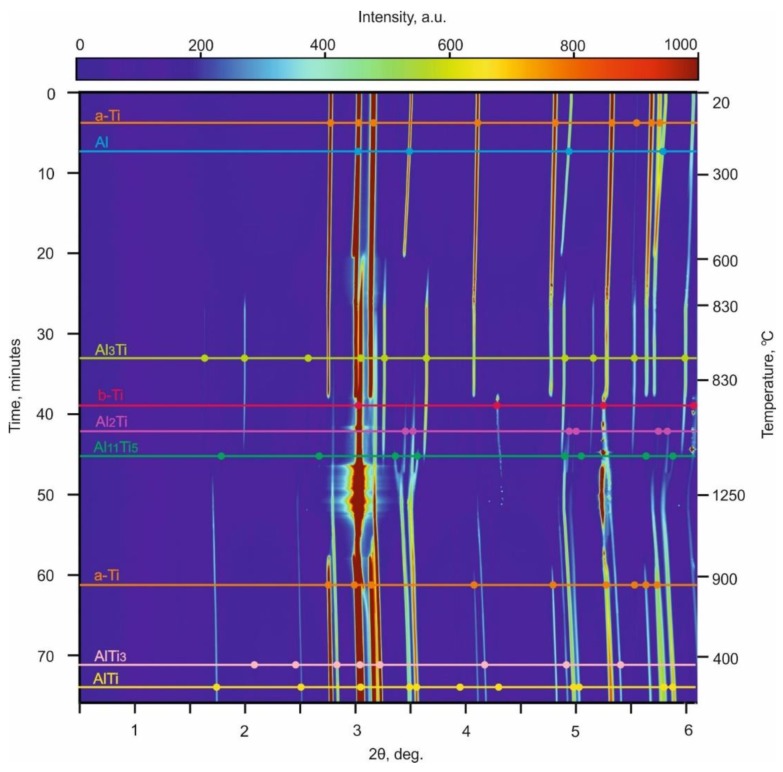
The synchrotron X-ray diffraction patterns represented in 2θ–temperature/time coordinates. Each row of the “map” corresponds to a one-dimensional diffraction pattern obtained at a particular temperature and time. The map illustrates the phase transformations that occur in the Ti–Al system during heating to 1250 °С.

The reactions between Ti and Al on heating are shown in Figure 3. Any point on the diagram represents the intensity of X-ray radiation at a particular 2θ angle and the corresponding time. In the initial state (before heating), the only significant X-ray intensity observed is for 2θ values associated with the hcp Ti and fcc Al phases. After the reactions were fully completed (i.e., after cooling down), the α_2_ -Ti_3_Al and γ-TiAl phases were present. The α-Ti peaks observed in Figure 3 after cooling correspond to the titanium container, which reacted only partially with a powder mixture during heating. The final intermetallic phases were formed via several reactions with the formation of intermediate phases. A detailed analysis of these reactions is given below.

**Figure 4:**
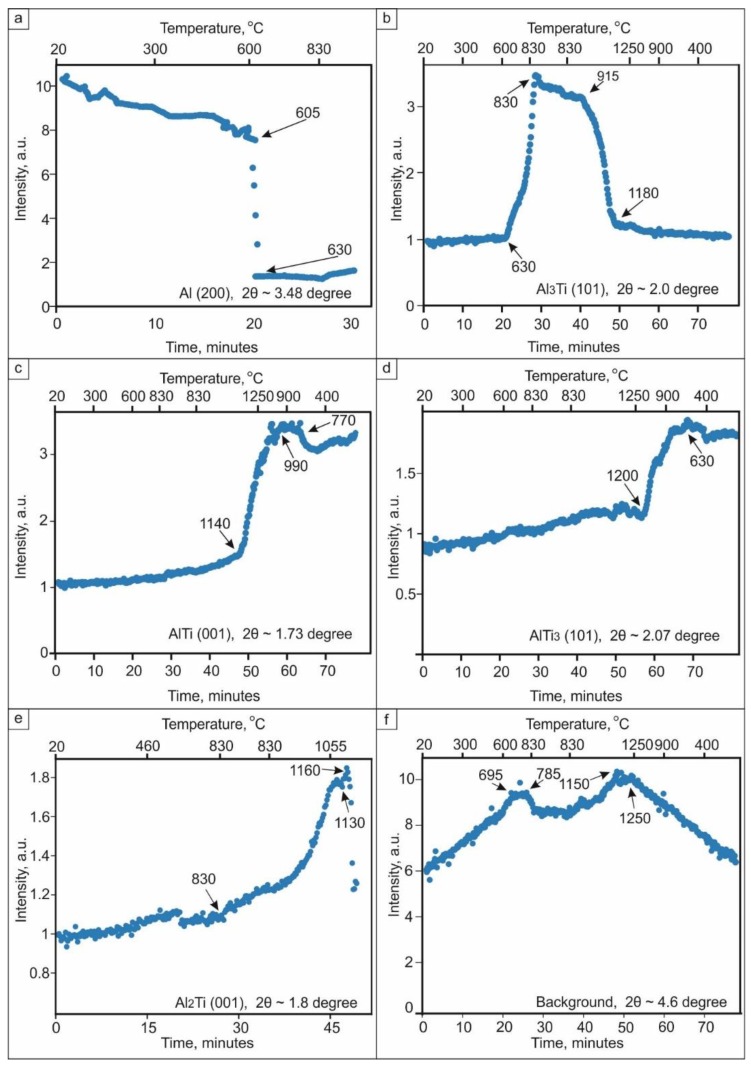
The increase in intensity of peaks of different phases during heating to 1250 °С: (**a**) intensity of Al (200) peak at 2θ = 3.48 degrees; (**b**) intensity of Al_3_Ti (101) peak at 2θ = 2.0 degrees; (**c**) intensity of AlTi (001) peak at 2θ = 1.73 degrees; (**d**) intensity of AlTi_3_ (101) peak at 2θ = 2.07 degrees; (**e**) intensity of Al_2_Ti peak at 2θ = 1.8 degrees; (**f**) intensity of a background at 2θ = 4.6 degrees.

The only change observed during the early stage of heating was a shifting of the Ti and Al peaks to lower angles due to the thermal expansion of the materials. Between 605 and 630 °С, the Al peaks disappeared, which can be clearly seen in Figure 4a. This happened due to the melting of Al and the beginning of a reaction between Al and Ti. The temperature of 630 °С corresponds to an intensive formation of Al_3_Ti. Titanium trialuminide peaks grew in intensity in the temperature range between 630 and 830 °С (Figure 4b). This compound existed up to 1180 °С. Further heating led to an interaction between the titanium trialuminide and titanium and the subsequent formation of Ti-enriched phases. The TiAl phase formed at 1140 °С (Figure 4с); Ti_3_Al peaks appeared around 1200 °С (Figure 4d).

**Figure 5:**
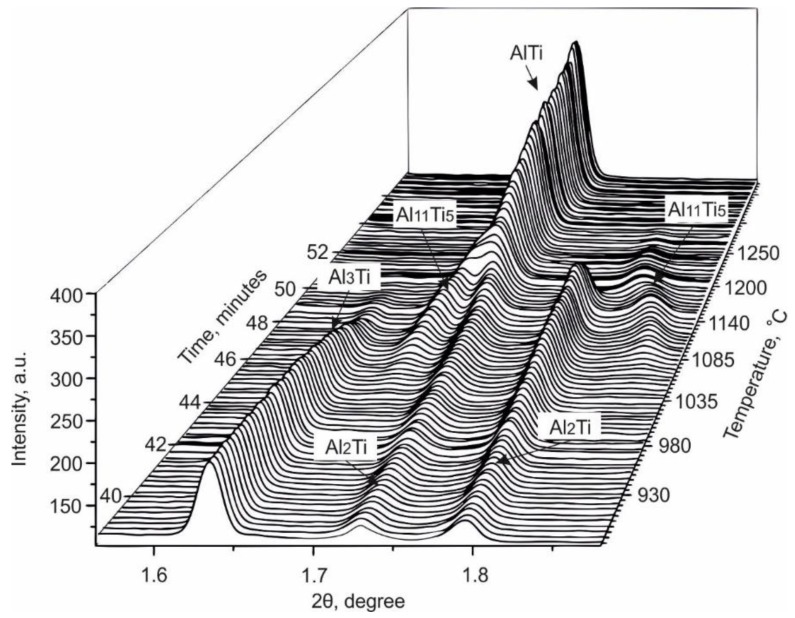
The formation of Al_11_Ti_5_ and TiAl phases during heating of the Ti and Al powder mixture observed by in situ synchrotron diffraction.

It should be mentioned that the formation of TiAl occurred via intermediate reactions (Figure 4e and Figure 5). Firstly, at a temperature of 830 °C, the intensity of titanium trialuminide peaks started to decrease as the formation of Al_2_Ti began. The latter phase existed up to a temperature of 1140 °C when TiAl nucleated. Over the temperature range between 1035 and 1200 °С, an Al_11_Ti_5_ intermediate compound appeared. This phase is known as the one-dimensional antiphase domain structure (1d-APS) [40] or “long-period structure” (LP).

The normal Ti α→β transformation can be observed in Figure 3; the reverse transformation occurs on cooling. During the cooling stage, the peaks of all phases shift to higher angles, indicating a decrease of the lattice parameters of the metallic and intermetallic phases due to thermal contraction.

**Figure 6:**
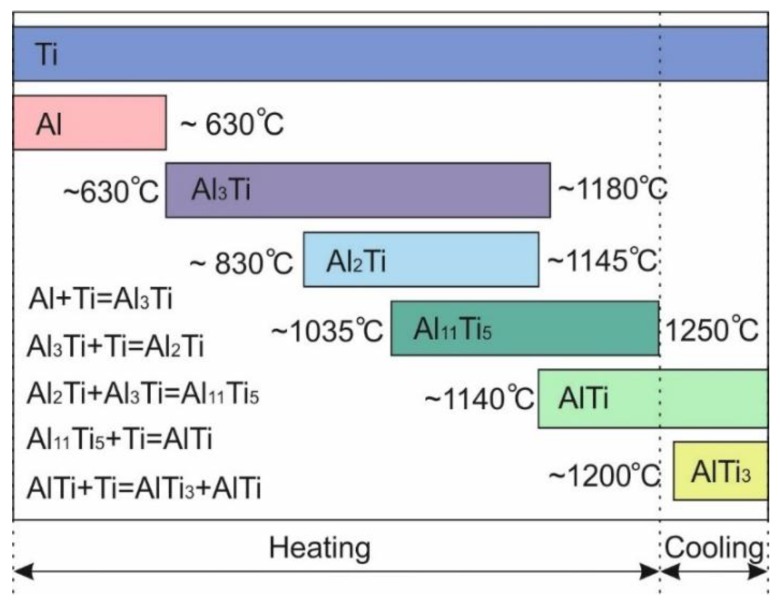
The sequence of the reactions in the binary Ti–Al system during heating from room temperature to 1250 °С and subsequent cooling.

The analysis of the obtained data, and consideration of the Ti–Al phase diagram, allowed us to determine the sequence of reactions occurring in the system during heating and choose the appropriate temperature for further reactive sintering of Al and Ti foils. The sequence of the reactions is shown in Figure 6. Being more thermodynamically stable, Al_3_Ti is the first compound to form. According to [38], the process of its formation continues until the Al is completely consumed. Since the intensity of the Al_3_Ti peaks increased up to 830 °С, it can be supposed that Al remained in the sample up to this temperature. However, even at 630 °С, it fully transformed to a liquid state. The early melting of Al can be explained by exothermal reaction of Al_3_Ti formation and the heat release, which initiate the transition of Al to a liquid state already at 630 °C. The formation of liquid Al can be approved by increase of the background intensity, which reaches the maximum at 695 °С and decreases in the temperature range between 785 and 830 °С (Figure 4f). Thus, holding the sample at 830 °С promotes the full consumption of Al during the formation of titanium trialuminide. Due to the lack of aluminum, the reaction between Ti and Al_3_Ti starts. Al_2_Ti is the product of this reaction. According to the Al–Ti phase diagram [40], at a temperature close to 1000 °С, the eutectoid mixture of Al_3_Ti and Al_2_Ti transforms to TiAl/1d-APS. First, the 1d-APS forms and then the nucleation of TiAl takes place. This phenomenon can probably be explained by the fact that Al_11_Ti_5_ forms directly from the reaction of Al_3_Ti and Al_2_Ti. However, the Ti content in TiAl should be higher than that in Al_3_Ti and Al_2_Ti, and consequently interaction of the aforementioned components with Ti to form this phase is required. Due to the wide stability range of the TiAl phase, its composition can widely vary. The content of Ti in TiAl can reach about 53 at.% at 1120 °С and drops slightly as the temperature decreases. For this reason, an increase of the Ti_3_Al intensity is observed when the sample is cooled: the excessive Ti in the TiAl phase aids the formation of the more Ti-rich α_2_ phase.

It can be concluded that the desirable (TiAl + Ti_3_Al) composition of the material can be obtained by heating the elemental foils to a temperature of 1250 °С, which guarantees the full completion of the reaction and the formation of the two (γ and α_2_) phase structure.

### 3.2. Characterization of Laminate Composites Obtained by the SPS of Elemental Foils and Ceramic Particles

#### 3.2.1. Characterization of Composite Structure

**Figure 7:**
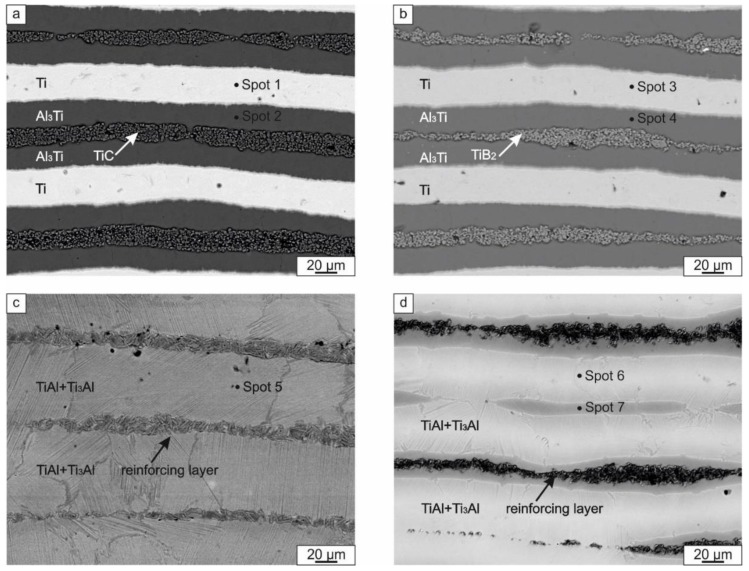
The cross section of the materials: (**a**) the composite reinforced by TiC after the first sintering step; (**b**) the composite reinforced by TiB_2_ after the first step of sintering; (**c**) the composite reinforced by TiC after the second step of sintering; (**d**) the composite reinforced by TiB_2_ after the second step of sintering. The images were taken using an SEM in the backscattered mode.

Figure 7a,b show the cross section of composites reinforced by TiB_2_ and TiC particles after the first stage of sintering (830 °C). As can be seen, the materials possessed a layered structure. EDX analysis revealed that the brighter layers were titanium and that the darker layers consisted of titanium trialuminide (Table 1). The reinforcement particles (TiB_2_ (Figure 7a) and TiC (Figure 7b)) were located between the dark-gray layers of Al_3_Ti.

**Figure 8:**
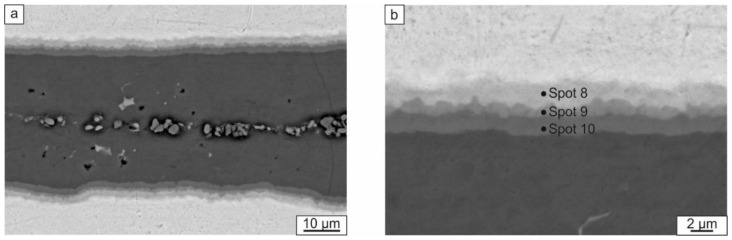
Al_3_Ti layer (**a**) and the transition layer between Al_3_Ti and Ti (**b**) developed during the SPS of specimens at 830 °C.

Besides the thick bright and dark layers, several thin interlayers were observed in the composites. They were formed at Ti and Al_3_Ti interfaces (Figure 8). Since the SEM images were recorded using the backscattered electron detector, the gradual change of colors within the layers indicates differences in the Al/Ti ratio. The brighter the layer, the higher its Ti content. The results of elemental analysis of these interlayers are shown in Table 1. The phenomenon of intermetallic interlayer formation during SPS of Ti–Al_3_Ti composite has been described previously [38]. EDX and TEM investigations revealed that the interlayers consisted of several phases: Al_2_Ti, AlTi, and AlTi_3_. Their formation resulted from the reaction between Ti and Al_3_Ti when Al was fully consumed.

**Figure 9:**
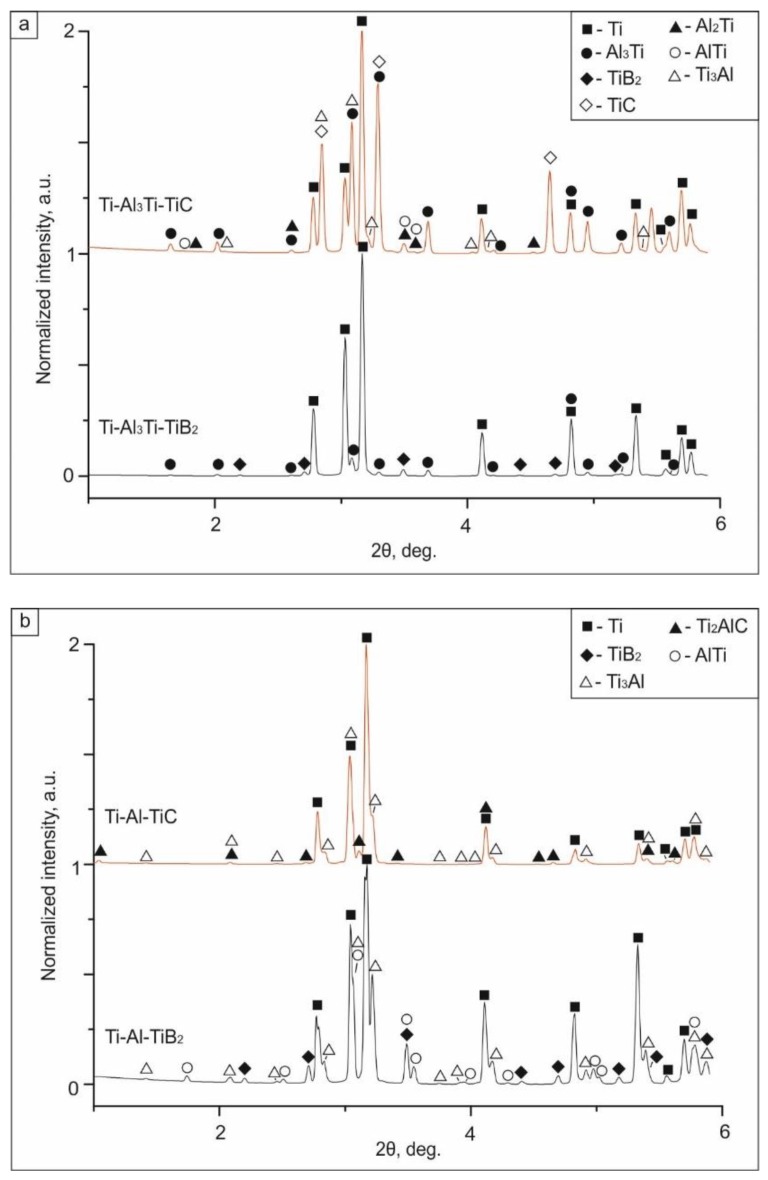
The phase composition of multilayer composites containing TiB_2_ and TiC after SPS at: (**а**) 830 °С; (**b**) 1250 °С. The highest peak intensity was 1000 arbitrary units.

X-ray diffraction analysis confirmed the formation of additional phases in the composites (Figure 9a). In the diffraction pattern of the sample with titanium carbide, the minor peaks corresponding to Al_2_Ti, AlTi, and Ti_3_Al could be identified. The major peaks corresponded to Ti, Al_3_Ti, and the ceramic component (TiB_2_ or TiC). Phases that resulted from transformations between reinforcing phases and the matrix elements during low-temperature sintering were not observed.

The formation of Ti-rich phases during SPS at 830 °C was observed in [38]. According to the above-described in situ synchrotron diffraction analysis, holding the samples at this temperature does not result in the formation of Ti-rich phases. Consequently, TiAl and Ti_3_Al compounds could appear as a result of local overheating during SPS induced by inhomogeneous heat distribution across the sample. Overheating could be explained by the particularities of the SPS process [41] as well as by a release of heat when the Ti and Al react [42].

**Figure 10:**
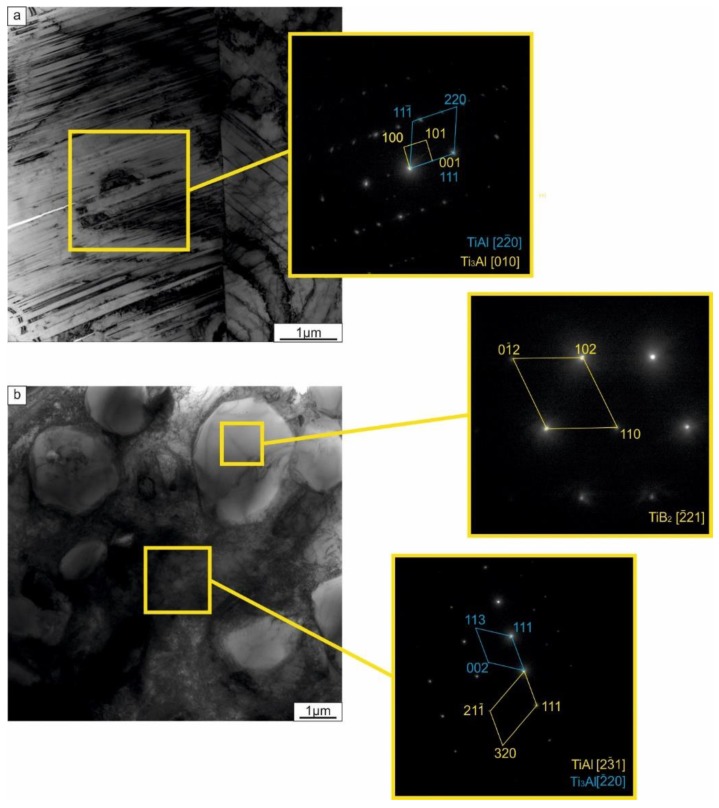
TEM micrographs of the sample reinforced with TiB_2_ particles: (**a**) an intermetallic layer; (**b**) a reinforcement layer.

During the second step of SPS, the samples were heated to a higher temperature (1250 °C) and kept at this temperature for 5 min. The microstructure of the resulting composites obtained is shown in Figure 7c,d. Both materials consisted of alternative layers of intermetallic and a ceramic phase. It should be mentioned that, in the composite reinforced by TiC, two types of layers were observed, while in the material containing TiB_2_, three types of layers were formed. Dark layers in the sample with TiB_2_ consisted of 51.8 at.% Al and 48.2 at.% Ti, i.e., had a composition similar to equiatomic TiAl. These layers were mainly located along the reinforcement inclusions and their presence can probably be explained by incomplete diffusion between titanium and titanium trialuminide. Elemental analysis of the light layers revealed that they consisted of approximately 37 at.% Al and 63 at.% Ti. According to the Al–Ti phase diagram, alloys in the concentration range between 33 and 50 at.% Al contain a mixture of γ and α_2_ phases. The two-phase structure of these layers was confirmed by microstructural analysis, which revealed the lamellar morphology of the intermetallic layer (Figure 7с,d). The lamellar structure was also observed using TEM (Figure 10). Analysis of the diffraction patterns obtained by TEM (Figure 10a) and X-ray diffraction analysis (Figure 9b) confirmed the existence of both the γ and α_2_ phases. The sample reinforced by TiC contained about 37 at.% Al in the intermetallic layer. The deficiency of Al in intermetallic layers can be explained by its interaction with the reinforcement phase. Structural analysis (Figure 7a,c) established that the morphology of TiC particles changed after 1250 °С sintering: equiaxed grains presented in the sample after 830 °С transformed into elongated grains. X-ray diffraction analysis of the sample reinforced with TiC confirmed that, during heating to 1250 °С, the TiC particles interacted with Al, which resulted in the formation of Ti_2_AlC (Figure 9b). According to an isothermal section of the ternary Ti-Al-C phase diagram, this phase exists at 1250 °C [43]. Ti_2_AlC is a typical example of M_n+1_AX_n_ (MAX) phases [44]. Thus, Al was partly consumed to the formation of the MAX phase, which prevented the formation of γ-phase in this sample. The absence of γ-TiAl is clearly seen in the XRD pattern in Figure 9b. During sintering, no interaction between the titanium di-boride and the matrix material occurred. This is apparent from the presence of unreacted TiB_2_ within the final microstructure (Figure 5b). In a number of studies [45,46,47], the process of TiB phase was observed by SPS of a TiB_2_ powder or (TiB_2_ + Ti) composites; however, the process of new phase formation requires the higher temperatures. It was also shown for the Ti-Al-TiB_2_ sample that the particles of the reinforcement phase did not form a solid layer but were densely distributed within a thin layer of the intermetallic matrix (Figure 10b). Diffraction patterns obtained from two neighboring regions indicated the co-existence of TiB_2_ and a (α_2_ + γ) mixture in the reinforcing layer.

#### 3.2.2. Mechanical Properties of the Materials

The composites obtained after SPS processing had very heterogeneous microstructures due to the presence various different layers. The properties of these layers differ significantly. To estimate the properties of individual layers, microhardness testing was applied.

The microhardness of the intermetallic layers was 427 ± 39 HV. The microhardness of the layers containing TiB_2_ and T_i2_AlC particles was significantly higher: 1555 ± 256 HV and 784 ± 83 HV, respectively. It is interesting to note that the microhardness of the Ti_2_AlC layer measured in this work was higher than that reported by other authors [48,49]. It was claimed in the aforementioned studies that the microhardness of pure Ti_2_AlC ranges from 3 to 6 GPa. The microhardness of TiB_2_ is usually 25–35 GPa. The lower microhardness level of the TiB_2_ layers obtained in the composite is due to the structure of these layer represented by the intermetallic matrix with hard ceramic inclusions. The same is evidenced by a wide range of interval of confidence. The distribution of hard particles is inhomogeneous, and the lower microhardness level (about 1200 HV) corresponds to a small density of particles at the surface area, while the higher density of the particles leads to the higher microhardness (up to 1900 HV).

**Figure 11:**
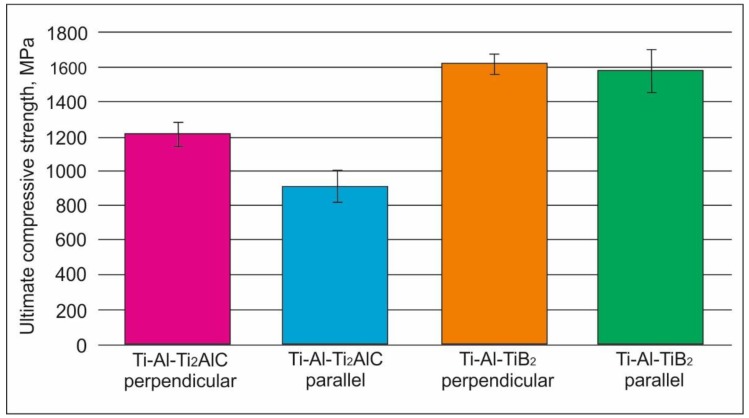
The ultimate compressive strength of the samples reinforced by TiB_2_ and Ti_2_AlC particles.

Compression tests were carried out to estimate the room temperature strength of the composites. The results of tests obtained when the load was applied either parallel or perpendicular to the layers are shown in Figure 11. The tests showed that the ultimate compressive strength of the samples containing TiB_2_ was higher compared to that of the composite reinforced with Ti_2_AlC. It may be noticed that the orientation of the layers is important only in the case of Ti_2_AlC reinforcement.

The highest compressive strength reached 1600 MPa for the material reinforced by TiB_2_ layers. According to the literature [50,51,52], the typical value of the ultimate compressive strength for the binary two-phase TiAl alloy equals to 1415–1430 MPa. Thus, addition of TiB_2_ contributes to the strengthening of a binary TiAl. At the same time, Ti_2_AlC is not efficient as a reinforcing phase. This observation is in good agreement with the data presented in [50,51], showing that if a composite contains only TiB_2_ particles as a reinforcing phase, the increase of the compressive strength can be more significant compared to the material reinforced by both TiB_2_ and Ti_2_AlC.

**Figure 12:**
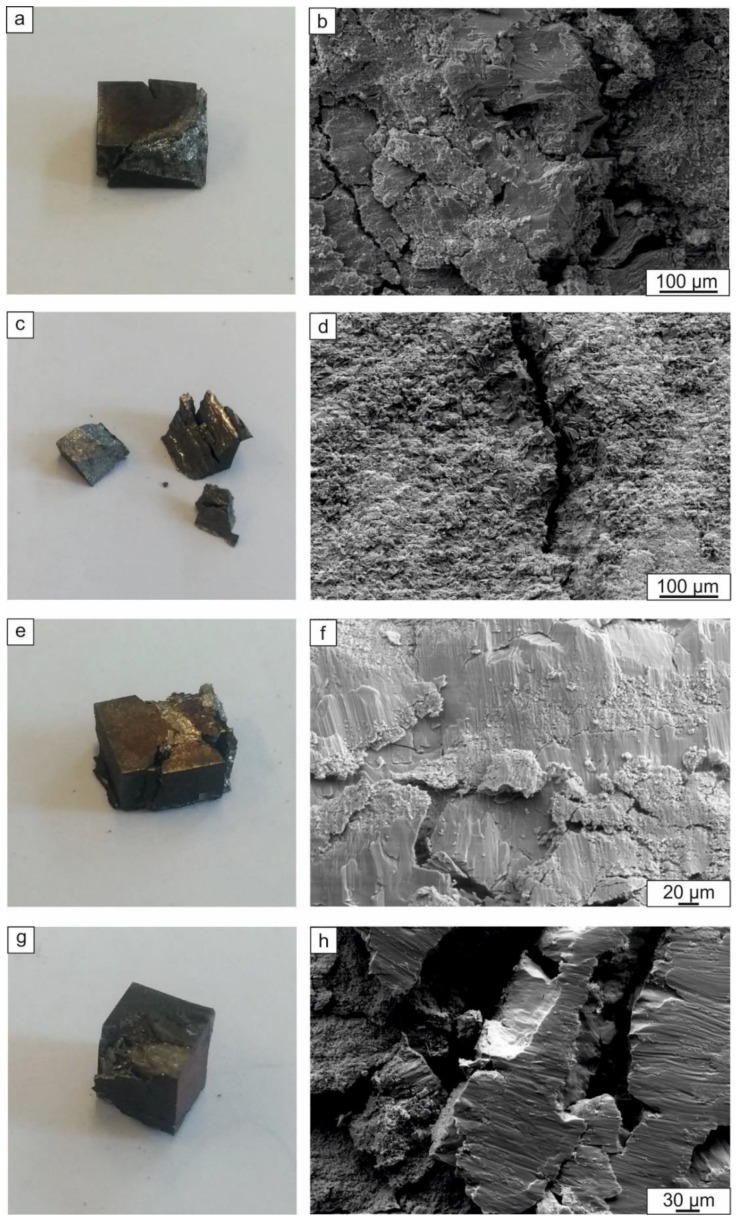
Fractographic investigations of the samples reinforced with Ti_2_AlC (**a**–**d**) and TiB_2_ (**e**–**h**) after compression testing parallel (**c**,**d**,**g**,**h**) and perpendicular (**a**,**b**,**e**,**f**) to the layers.

Fractographic investigations of the samples after the compressive tests showed that loading the materials parallel to the layers mainly caused a delamination type of fracture (Figure 12c). Loading perpendicular to the layers led to an upsetting of the sample and nucleation of cracks in the direction of loading (Figure 12a,e). A specific feature of the composite reinforced with TiB_2_ was a sliding-type of fracture, where the layers slid relative to one another and left the traces at the fracture surface (Figure 12f,h). These traces were probably formed by the movement of reinforcing particles at the interfaces between two adjacent intermetallic layers. Fracture of the samples containing Ti_2_AlC occurred in a brittle manner, resulting in an intergranular fracture surface (Figure 12b,d).

The difference in strength between the two testing directions can be probably explained by the shearing processes that took place when the specimens were loaded parallel to the layers (Figure 12d). The presence of the reinforcement layers does not allow the sample to be freely deformed when the load is parallel to the layers (Figure 12a,e) when mainly the intermetallic component of the composite is subjected to deformation. Thereby, the strong ceramic layers in parallel mode restrict deformation and lead thus to premature brittle failure.

**Figure 13:**
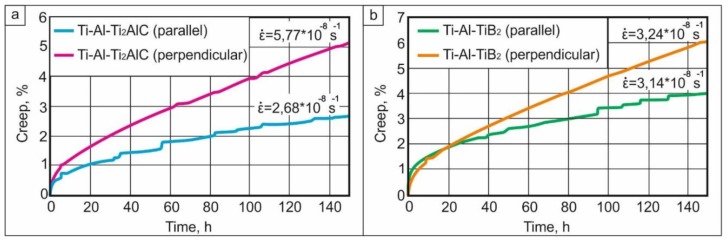
The creep curves for tests performed at 750 °С at an initial stress of 250 MPa: (**а**) samples reinforced with Ti_2_AlC; (**b**) samples with TiB_2_.

**Figure 14:**
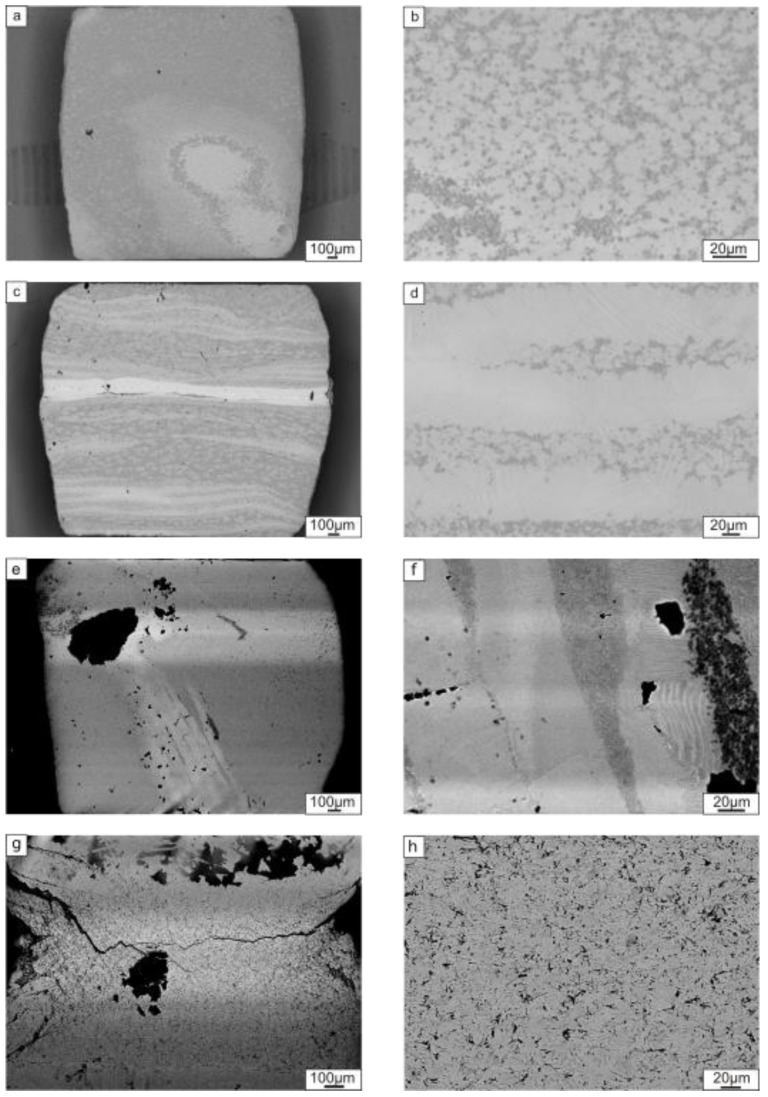
The microstructure of the composite reinforced with Ti_2_AlC after creep testing to around 150 h: (**a**,**b**) loading parallel to the layers; (**c**,**d**) loading perpendicular to the layers; and the composite reinforced by TiB_2_ after creep testing to around 150 h; (**e**,**f**) loading parallel to the layers; (**g**,**h**) loading perpendicular to the layers.

The creep tests revealed that loading parallel to the layers resulted in better creep resistance compared to samples loaded perpendicular to the layers (Figure 13). The layers containing ceramic particles seem to have acted as reinforcing elements and did not allow the sample to be easily deformed. Thus, the creep strain of the samples tested perpendicular to the layers was higher (Figure 14a,b). After 150 h of loading at 750 °C perpendicular to the layers, creep strains of 5.2% and 6% were developed in the Ti_2_AlC- and TiB_2_-reinforced composites, respectively. For loading parallel to the layers, the creep strain values were 2.7% and 4%, respectively. For both composite types, the creep strain developed was lower for loading parallel to the layers. However, in the case of TiB_2_ reinforcement, the difference in the strain rate values was insignificant.

The creep rate did not vary significantly as a function of the type of reinforcing particles and a scheme of loading. In all cases, it was of the order of 10^−8^ s^−1^. A small anisotropy was observed when loading the material with Ti_2_AlC layers: the specimens loaded in the direction parallel to the layers possessed a lower creep rate. Thus, the presence of a hard phase contributes to the creep properties of TiAl alloy independently of their composition or orientation. Reinforcement with the ceramic phase slows down creep by 2–3 orders of magnitude depending on the structural state of the alloy. It was reported in [53] that the Ti-Al48 alloy with equiaxed grains demonstrated a creep rate of about 9 × 10^−5^ s^−1^, while the alloy with the lamellar structure possessed the creep rate of 9 × 10^−5^ s^−1^ under the same loading and temperature conditions chosen in this work. The close value of the magnitude for a creep rate as that demonstrated in the present study was reached by the complex alloying of TiAl. For example, the value of 10^−8^ s^−1^ was observed for Ti-47Al-2Cr-2Nb [54,55], Ti-48Al-2Cr-2Nb-1B [56], Ti-46Al-2W-0.5Si-B [56], and Ti-46.0Al-1.8Cr-3.0Nb-0.2W-0.1C-0.2Si [53].

The metallographic investigation of the crept specimens reinforced with Ti_2_AlC showed that the layered structure was better preserved when loading perpendicular to the layers (Figure 14c,d). For loading parallel to the layers fragmentation of the layers was observed (Figure 14a,b). Particles of the reinforcement phase formed agglomerates in the intermetallic matrix. Investigations of the samples containing TiB_2_ revealed weak traces of the layers when loaded in the direction parallel to the layers (Figure 14e,f). No layered structure was preserved during creep testing perpendicular to the layers (Figure 14g,h). Due to the high hardness, the sample subjected to cracking when loaded.

**Figure 15:**
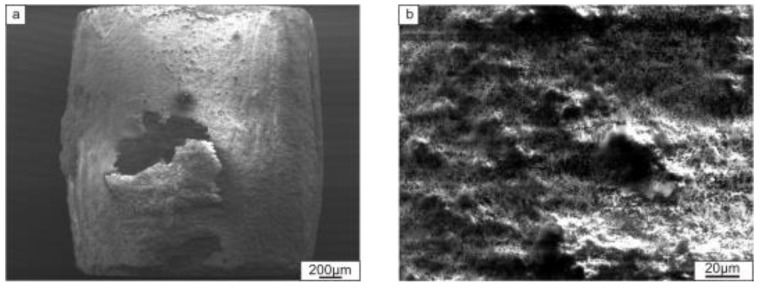
The surface of the composite reinforced with Ti_2_AlC after creep testing (**a**) and the structure of an oxide layer (**b**).

Since the creep tests were performed in the air atmosphere, formation of an oxide layer was found on the surface of all samples (Figure 15).

## 4. Conclusions

1. The sequence of phase transformation leading to the formation of a two-phase (α_2_ + γ) structure from the reaction of Ti and Al was studied using in situ synchrotron X-ray diffraction analysis. The formation process occurred via a series of liquid–solid and solid–solid reactions and was accompanied by the formation of a number of intermediate phases. Some of these intermediate phases (e.g., Al_11_Ti_5_) were preserved in the material until the final stage of sintering at 1250 °С. The precipitation of Ti_3_Al occurred during cooling.
2. Heating the ceramic particle containing laminated samples to 1250 °С resulted in reactions between the metallic components themselves and also with the reinforcement particles (in the case of TiC reinforcement). For the composite made with TiC, a reaction between the TiC and the metallic elements produced a Ti_2_AlC MAX phase. At the same time, the reaction between ceramic particles and the matrix in the sample reinforced by TiB_2_ was not observed. TiB_2_ particles did not form a solid layer; TEM investigations revealed that the reinforcement layers consisted of TiB_2_ within an intermetallic matrix.
3. Compression tests showed that the strength of the material reinforced by TiB_2_ was higher than that of the Ti_2_AlC (TiC-based composite) material. The relation between orientation of the layers with respect to the loading direction and mechanical properties was observed only in the case of Ti_2_AlC reinforcement. The room temperature strength was higher when loading was perpendicular to the layers. However, this loading orientation had worse creep resistance at 750 °C/250 MPa than when loading was performed parallel to the layers.

## Figures and Tables

**Table 1:** EDX analysis of the local areas shown in Figure 7 and Figure 8b.

Spot Number	Al (at.%)	Ti (at.%)
Spot 1	-	100
Spot 2	74.75	25.25
Spot 3	-	100
Spot 4	74.4	25.6
Spot 5	37.3	62.7
Spot 6	36.9	63.1
Spot 7	51.8	48.2
Spot 8	12.8	87.2
Spot 9	44.1	55.9
Spot 10	63.5	36.5
